# Case Report with Systematic Literature Review on Vascular Complications of BCG Intravesical Therapy for Bladder Cancer

**DOI:** 10.3390/jcm11206226

**Published:** 2022-10-21

**Authors:** Brianna King, Dhanveer Singh, Animesh Rathore, Ronald Flenner, Mark Flemmer

**Affiliations:** 1 Eastern Virginia Medical School, Norfolk, VA 23507, USA; 2 Department of Internal Medicine, Eastern Virginia Medical School, Norfolk, VA 23507, USA; 3 Department of Vascular Surgery, Sentara Norfolk General Hospital, Norfolk, VA 23507, USA; 4 Department of Infectious Disease, Eastern Virginia Medical School, Norfolk, VA 23507, USA

**Keywords:** BCG, Bacillus Calmette-Guerin, *Mycobacterium*, aneurysm, mycotic aneurysm, bladder cancer, vascular complications, vascular abnormalities

## Abstract

(1) Background: Intravesical instillation of therapeutic Bacillus Calmette-Guerin (BCG) is the standard of treatment for non-muscular invasive bladder cancer. Although the exact immunomodulatory effects of BCG therapy in non-muscular invasive bladder cancer (NMIBC) are still unclear, it has been considered a safe and effective treatment with the largest to-date report of complications citing minimal side effects, none of which included arterial involvement; (2) Methods: A systematic literature review was performed using PubMed, Cochrane, Medline, and Google Scholar from database inception to March 2021. Only eligible studies reporting aneurysm formation in adult patients with a history of BCG immunotherapy and no previous vascular pathology were included; (3) Results: A systematic literature review was conducted, highlighting 17 reports suggestive of BCG-induced mycotic aneurysm development. We added a case of a 78-year-old male, 30 months after last BCG-instillation, with a mycotic abdominal aneurysm yielding *Mycobacterium tuberculosis* with pyrazinamide resistance culture.; (4) Conclusions: Concluding results suggest a higher incidence of vascular complications from BCG intravesical therapy in the treatment of non-muscular invasive bladder cancer than previously reported. Recommendations are made to emphasize further research of this immunotherapy complication to facilitate the creation of guidelines for diagnosis and management of these patients.

## 1. Introduction

Bacillus Calmette-Guerin (BCG) has been used as immunization against *Mycobacterium tuberculosis* since its development in the early 1900s [1]. Its therapeutic effects have been widely studied, including its currently accepted use in treatment of superficial bladder tumors [2,3]. In 1992, Lamm et al. reported a five percent incidence of serious side effects in patients treated with intravesical BCG, none of which included vascular complications [4]. Recent reports suggest BCG-induced mycotic aneurysm as a complication of immunotherapy although it is still considered rare [5]. Mycotic aneurysms are considered a life-threatening vascular pathology with reported perioperative mortality of 26–44% [6]. Although operative repair and antibiotic therapy is the accepted treatment, there is current debate regarding superiority of open versus endovascular repair [7]. In this systematic review, we compile literature reports of vascular complications, specifically aneurysms, thought to be related to intravesical BCG therapy for the treatment of NMIBC. Authors add a reported case of de novo mycotic aortic aneurysm after multiple BCG intravesical treatments for NMIBC. The primary aim of this article is to compile current reports in literature of vascular complications of BCG intravesical therapy for bladder cancer.

## 2. Case

**Figure 1:**
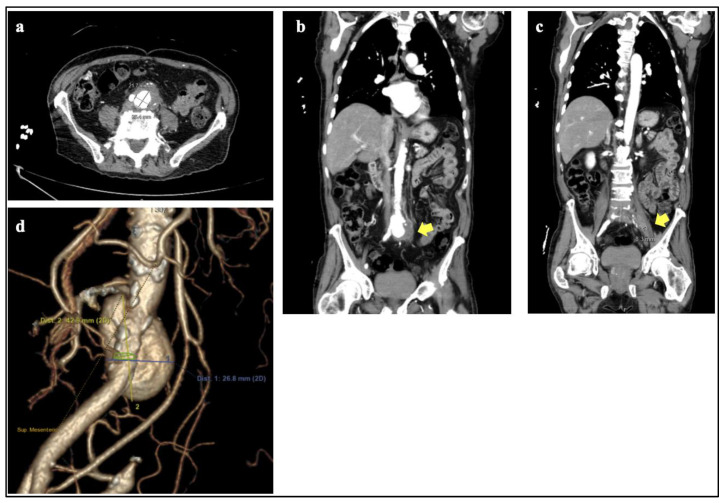
Mycotic aneurysm of abdominal aorta (**a**) Axial (with measurements) and (**b**) coronal images of periaortic soft tissue density surrounding saccular aneurysm. (**c**) Coronal image of periaortic soft tissue density extending into medial left psoas muscle. (**d**) CTA 3D reconstruction of saccular aneurysm with measurements.

We present the case of a 78-year-old male with a medical history of urothelial carcinoma of the bladder treated with nine months of BCG in 2018, coronary artery disease, diabetes mellitus, hypertension and hypercholesterolemia. BCG treatment for urothelial carcinoma of the bladder was complicated by two hospitalizations attributed to urinary tract infection (October 2018) and severe sepsis vs. BCG induced cytokine storm (December 2018). Urine and blood cultures were negative for acid fast bacilli (AFB). In August 2020, the patient was diagnosed with TaN0M0 high grade urothelial carcinoma of the bladder tumor recurrence and underwent cystourethroscopy and TURBT with gemcitabine 4 weeks later. At a follow up appointment, an annual CT urogram was completed with notable findings of L5-S1 inflammation and abscess with involvement of the left psoas muscle, abdominal aortic bifurcation, and proximal iliac arteries. The patient presented to the ED several days later with back pain, suprapubic pain and weakness. A CT angiogram was completed confirming discitis/osteomyelitis at L5-S1, paravertebral soft tissue inflammation at L4-S1 with periaortic infectious process extending into the left psoas muscle, IMA (inferior mesenteric artery), bilateral common iliac arteries, and complete occlusion of the left common iliac and left external iliac arteries, and associated 4.2 cm mycotic aortic aneurysm that was not seen previously (Figure 1). MRI of the lumbar spine further defined the aneurysm as 4.7 × 3.8 cm infrarenal abdominal aortic mycotic aneurysm. The patient underwent an open repair with discovery of a contained aneurysm rupture into the retroperitoneum. Intraoperative cultures were collected and a cryopreserved aortoiliac artery was utilized for reconstruction. Intraoperatively the patient had multiple occurrences of hemodynamic instability with progression to pulseless electrical activity resulting in death. Intraoperative blood cultures showed no growth at five days and initial AFB smear was negative. AFB culture of aortic aneurysm tissue grew pyrazinamide resistant *Mycobacterium tuberculosis* 25 days later.

## 3. Materials and Methods

**Figure 2:**
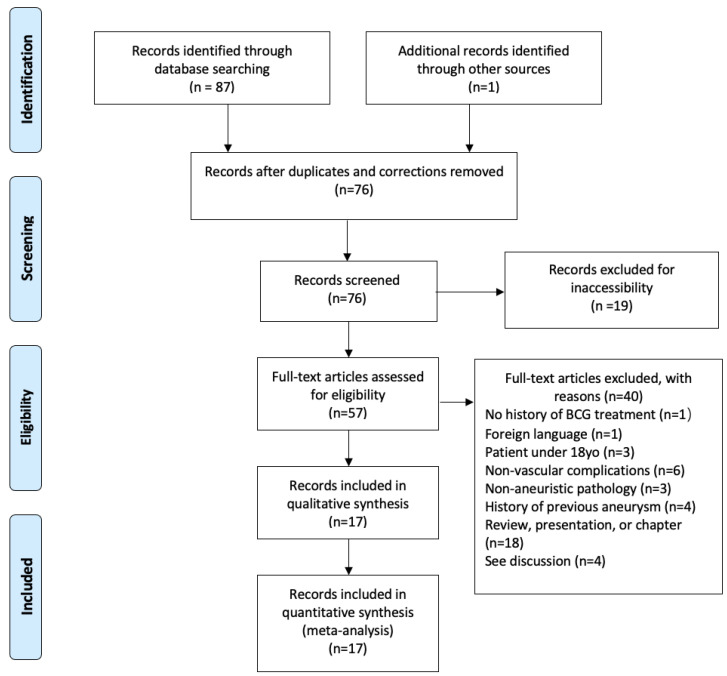
PRISMA (Preferred Reporting Items for Systematic reviews and Meta-Analyses) diagram of case report selections.

We performed a systematic literature review identifying reported cases of first occurrence aneurysm and pseudoaneurysms in adult patients with a previous history of BCG intravesical therapy. Authors decided to limit search parameters to adults (18 and older) exposed to Bacillus Calmette-Guerin during intravesical treatment of non-invasive muscular bladder cancer as bacillus Calmette-Guerin in the context of children is almost exclusively utilized for vaccination. This review followed PRISMA guidelines and was limited to English-language reports with no limitation on publication year. A total of three queries were created: (“BCG” AND “aneurysm”) was used to search Pubmed and Cochrane, (“BCG” AND “aneurysm” AND “bladder cancer”) was used to search Medline (OVID), and (“BCG” AND “mycotic aneurysm” AND “bladder cancer”) was used to search Google Scholar. On 14 August 2021, a multidatabase literature search was performed utilizing these queries. A total of 87 records were identified with publication dates ranging from 1993 to March 2021. A total of 76 records, including our case as reported above, were screened through this initial search once removing duplicates and publication corrections. It is notable that 19 of these screened records were inaccessible to our researchers. These records were excluded as this review is meant to encompass all published literature that is accessible to clinicians in multiple specialties as the identified patient population can present among a variety of specialties. Including our patient’s case, a total of 17 records comprising 18 case reports were identified as suitable to our report parameters after filtering results applying the inclusion criteria: (1) confirmed history of BCG intravesical therapy; (2) report available in English; (3) patient at or above the age of 18; (4) reported aneurysm/pseudoaneurysm vascular complication; (5) no previous history of aneurysm; (6) report was not presented in a review, presentation, or chapter format (Figure 2). Writers decided to exclude 4 additional reports due to deficiency in case-specific information that would allow researchers to confirm BCG immunotherapy treatment before aneurysm presentation.

## 4. Results

Among the 17 reports identified in our systematic review, and the additional case we report in this article, a total of 18 cases were identified [8,9,10,11,12,13,14,15,16,17,18,19,20,21,22,23]. Notable clinical characteristics extracted from these literature reports were patient’s age and gender, aneurysm location, extravascular involvement, time between most recent BCG instillation and admission, number of instillations, culture site, culture results, surgical treatment, discharge therapy, and reported outcomes (Table 1). Of these cases, all patients were men with a median age of 74.2 years. Each patient received intravesical immunotherapy before aneurysm presentation, of those with detailed treatment history reported, number of treatments ranged from 5 to 18 BCG instillations. Time from last intravesical treatment to admission was a median time of 10 months. Vascular involvement of the aorta was noted in 15 cases (83.3%) with the remainder of cases reporting aneurysm exclusively of the ulnar artery (5.5%), pseudoaneurysm of the common femoral artery (5.5%), or a combination of vascular involvement of the popliteal, common femoral, and iliac arteries (5.5%). A total of 17 of the 18 cases reported presence of either *Mycobacterium bovis*, BCG strain-specific *Mycobacterium bovis*, or *Mycobacterium tuberculosis*. These culture results were collected and processed per each medical treatment team’s discretion. It is important to note that in the individual case with no reported positive culture, no culture was obtained, although authors report high suspicion of mycotic aortic aneurysm with subsequent *M. bovis* graft infection [11]. All cases reported a surgical treatment to remove and/or repair infected vasculature. A total of 13 patients received varying antimycobacterial pharmaceutical therapy upon discharge whereas five patients received no treatment due to death or discontinuation of medical.

## 5. Discussion

Recent literature describing systemic vascular involvement after BCG treatment, specifically BCG-induced mycotic aneurysms, has been limited. BCG-induced mycotic aneurysm as a vascular complication of BCG therapy is rarely reported likely due to several reasons. Firstly, the patient population of bladder cancer often overlaps with those at high risk for aneurysm development: older, male smokers. Authors speculate underreporting of BCG induced aneurysm formation as it is a difficult diagnosis to support. Providers do not likely attribute aneurysm formation to iatrogenic causes and often a high degree of clinical suspicion is needed although uncommon in the typical patient presentation as those reviewed here. Secondly, mycotic aneurysm formation is more often found at sites of previous vascular injury, thus high incidence of graft infection. Our review focused on reports of patients with no previous vascular pathologies to examine the current literature regarding diagnosis and treatment strategies of patients presenting with BCG-induced mycotic aneurysms. Lastly, the mechanism of BCG induced mycotic aneurysm formation is unclear warranting further study however authors might propose BCG reactivation by an unknown cause as all patients presented with symptoms after multiple treatments of intravesical therapy. It is premature to form speculations of potential risk factors due to limited reports of such occurrences, as demonstrated in this article.

It is important to obtain a detailed patient history, including previous BCG therapy. In the absence of any suspected mycobacterium exposure, a high clinical suspicion should be held for BCG-related complications. In our newly reported case, the patient presented with image-identified aneurysm findings years after his last BCG intravesical treatment, suggesting a need for extended follow up in these patients. Although this patient’s initial vascular presentation was first identified by annual urologic monitoring, CT angiography and MRI were required to establish a definitive diagnosis. It is also notable to mention that prior imaging in this patient was unremarkable, suggesting either reactivation of previously latent infection or exponential growth of mycobacterium since previous scans. Our patient’s culture results were initially reported as sterile, however growth of *Mycobacterium tuberculosis* with pyrazinamide resistance was reported 25 days later. This suggests that in circumstances of immediate negative culture results, BCG strain related *Mycobacterium* infection cannot be excluded. Positive culture results of *Mycobacterium bovis* can often be delayed for up to 12 months, as per patient presentation in Wolf et al. [23]. Initiation of anti-mycobacterial pharmacotherapy should be considered in cases where there is a high clinical index of suspicion for BCG-induced mycotic aneurysm.

There are no current guidelines for surgical or pharmacological management of BCG-induced mycotic aneurysm as it is a considerably new complication of BCG therapy noted in the literature. This systematic review, with additional case report presented by authors, attempts to compile a comprehensive list of accessible literature reports of aneurysms as a vascular complication of previous BCG intravesical therapy in the treatment of non-muscular invasive bladder cancer. Further research is necessary to explore long term vascular complications of BCG intravesical therapy in NMIBC treatment as it can aid clinicians in identifying and treating patients with vascular complications of BCG therapy such as BCG-induced mycotic aneurysms.

## 6. Conclusions

We present a literature review of 17 cases and an additional reported case suggestive of BCG-induced formation of mycotic aneurysms. Recently considered a rare complication of BCG immunotherapy, the incidence may be higher than the available literature suggests. Clinical recommendations are to consider a previous history of BCG intravesical therapy of NMIBC treatment in patients presenting with mycotic aneurysm formation in the absence of other identifiable risk factors. Clinicians should maintain a high index of suspicion under certain circumstances recognizing that confirmatory culture data is often delayed. Continued efforts should be made to expand research of the vascular complication of BCG immunotherapy as no guidelines of management exist at this time.

## Figures and Tables

**Table 1:** Summary of results from featured case reports of suspected BCG intravesical therapy induced aneurysms.

Case	Age/Sex	Aneurysm Location	Extravascular Spread	Months from Last Tx to Admit	# of Instillations	Cx Site	Cx Results	Surgical Treatment	Discharge Therapy	Reported Outcomes
1	73M	Thoracic AAA	Retrocrural/ posterior mediastinum collection	6	5	Retrocrural abscess	*M. bovis*	TEVAR and percutaneous drainage	RIPE +B6	Asymptomatic at 6 mo
2	67M	AAA	Infrarenal periaortic collection	4	15	AAA	*M. bovis*	Open repair with autologous graft	RIPE +B6	Asymptomatic at 6 mo
3	76M	Thoracic AA and AAA		12	NR	AAA AFB stain, T-RT, and DnaSP	BCG strain *M. bovis*	TEVAR and open repair with prosthetic graft	RIE	Asymptomatic at 6 mo
4	80M	Thoracic AA	Infiltrative lung consolidation	10	8 at 80 mg	Sputum PCR	*M. bovis*	TEVAR	RIE	No recurrence at 24 mo
5	70M	AAA		3	NR	NA	NA	EVAR	NR	Graft-site aortoenteric fistula at 10 d postop, critical colic ischemia with total colectomy, and unspecified death at 25 d
6	72M	R UAA		NR	9	R UAA	*M. tb*	Open repair with autologous graft	Ethambutol 9 mo	NR
7	66M	AAA	PE and R psoas muscle hematoma	NR	6	AAA PCR & FISH + ZN	BCG strain *M. bovis*, *M. tb*	“Surgical flattening of the aneurysm”	RIE 2 mo then RI 8mo	Aneurysm size reduction at 6 mo
8	73M	AApA	L iliopsoas	14	6	AApA	BCG strain *M. bovis*	Open repair with bovine graft	RIE 3 mo then RE + moxifloxacin	Postop severe inflammatory response syndrome, renal insufficiency, and chylous ascites treated w/HD, TPN, paracentesis, and octreotide; mycobacterium fluid collection at graft and left psoas muscle at 3 mo
9	80M	L CFApA		8	NR	L CFApA PCR	BCG strain *M. bovis*	Open repair with autologous graft	RIE 9 mo	NR
10	76M	AAA		NR	“biannual infusions for the prior 2 years”	AAA DNA probe	*M. tb*	Open repair with bypass	RIE (rifampin switched to rifabutin at 6 wks)	Pneumonia, sepsis, death at 4 mo
11	70M	AAA		2	18	AAA PCR	*M. bovis*	EVAR	NA	Aortic leak and splenomegaly treated with aortic repair, resection of para-aortic lymph nodes, and splenectomy at 10 mo, aortic rupture to death 1w postop
12	71M	AApA	Paraspinal muscles, retroperitoneum, and L4 & 5 ventral bodies	NR	NR	AApA PCR	*M. tb* RNA, BCG strain *M. bovis*	Open repair with nonautologous biologic graft	RIE 12 mo (ethambutol held for 1 mo)	Chills, night sweats, back pain and weight loss resolving 3 mo
13	81M	AAA		12	NR	AAA	BCG strain *M. bovis*	Open repair with bypass	NA	Aspiration-induced respiratory failure, death
14	86M	AAA	L psoas & retroperitoneum	NR	NR	L Psoas abscess	*M. bovis*	EVAR	NR	Renal failure to hospice
15	63M	L popliteal, L & R CFAA, and R CIAA		7	16	CFAA AFB	*M. bovis*	L pop stent, R CIAA EVAR with stent graft and coil embolization, and open CFA repair with graft	RIE 9 mo	Bilateral groin wound washout and VAC at 2 mo, LAD aneurysm with DNR at 4 mo
16	75M	AAA and L SFAA		14	5	AAA ZN and L SFAA	AFB, BCG strain *M. bovis*	Open repair with dacron graft bypass	RIE 12 mo	SFA hematoma and para-grafts granulation tissue tx: percutaneous SFA drainage at 1 mo, PAs at both grafts and R CIApA tx: SFA stent, AApA bypass and graft removal, and oversewing RCIA at 4 mo
17	80M	AAA	R psoas muscle	NR	7	AAA DNA probe (12 mo)	BCG strain *M. bovis*	Open repair with dacron bifurcation graft	RIE at 12 mo for 20 mo	Acute MI tx: PTCA, stent, and CABG at 5 mo; Para-graft R psoas fluid collection at 12 mo; graft-enteric fistula tx: graft removal and at 20 mo
Our Case	78M	AAA	L psoas abscess	30	9	AAA (25 d)	*M. tb* with pyrazinamide resistance	Open repair with allograft	NA	Cardiac arrest leading to death intraoperatively

AAA: abdominal aortic aneurysm, AA: aortic aneurysm, CFApA: common femoral artery pseudo-aneurysm; CFAA: common femoral artery aneurysm; CIAA: common iliac artery aneurysm; SFAA: superficial femoral artery aneurysm; TEVAR: thoracic endovascular aortic repair; EVAR: endovascular aortic repair; RIPE: rifampicin, isoniazid, pyrazinamide, and ethambutol; ZN: Ziehl-Neelsen; and PTCA: percutaneous transluminal coronary angioplasty.

## Data Availability

Not applicable.
